# Stimulator of interferon genes (STING)‐activating nanomedicines: Translating innate immune modulation into effective therapy for triple‐negative breast cancer

**DOI:** 10.1002/ctm2.70580

**Published:** 2026-01-05

**Authors:** Harshita Singhai, Taha Alqahtani, Humood Al Shmrany, Garima Gupta, Umesh Kumar Patil, Amirhossein Sahebkar, Prashant Kesharwani

**Affiliations:** ^1^ Department of Pharmaceutical Sciences Dr. Harisingh Gour Vishwavidyalaya (A Central University) Sagar Madhya Pradesh India; ^2^ Department of Pharmacology College of Pharmacy King Khalid University Abha Saudi Arabia; ^3^ Department of Medical Laboratory College of Applied Medical Sciences Prince Sattam Bin Abdulaziz University Al‐Kharj Saudi Arabia; ^4^ Graphic Era Hill University Dehradun India; ^5^ School of Allied Medical Sciences Lovely Professional University Phagwara Punjab India; ^6^ Biotechnology Research Center Pharmaceutical Technology Institute Mashhad University of Medical Sciences Mashhad Iran

**Keywords:** cuproptosis, ferroptosis, nanoparticle drug delivery, stimulator of interferon genes (STING) pathway, triple‐negative breast cancer

## Abstract

Triple‐negative breast cancer (TNBC), marked by profound immunosuppressive complexity, poses a critical challenge in therapy due to the absence of hormone receptors in its phenotype, making it unavailable for conventional therapies. The stimulator of interferon genes (STING) pathway is emerging as critical pathway translating the immunogenic ‘cold’ TNBC tumour into ‘hot’ one, thereby improving the responsiveness to immune checkpoint blockade (ICB). However, the clinical translation is still hindered by insufficient cytosolic delivery, rapid systemic degradation and tumour microenvironment‐induced metabolic inactivation. This review outlines the recent advances in STING‐mediated nanoparticle delivery with special emphasis on biomimetic, Trojan horse logic gate, manganese‐based and redox‐responsive stimuli delivery systems. Mechanistically, it integrates immune activation by ferroptosis, cuproptosis and mitochondrial DNA disruption. They synergise the amplification of type 1 interferon with dendritic cell maturation, potentiating antitumour immunogenesis. Notably, the combination with ICBs will further amplify the therapeutic potential of nanoparticles. Convergence of immunology and targeted therapies with nanoparticles opens new array for TNBC treatment. The review visualizes the clinical translation of mind maps into clinical reality, activating the innate immunity.

**Highlights:**

- STING activation converts immunologically cold TNBC into ICB‐responsive hot tumors.
- Nanoparticles overcome poor delivery, degradation, and TME‐driven STING inactivation.
- Biomimetic and stimuli‐responsive systems enhance type I IFN and DC maturation.
- Synergy with ICBs boosts innate immunity and antitumor immunogenesis.

## Graphical abstract

Graphical representation of STING‐activated nanomedicines is for effective treatment of triple‐negative breast cancer.

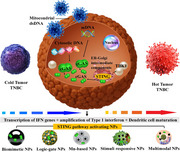

## 1 INTRODUCTION

Oncological pathology continues to own a critical challenge to global health, marked by uncontrolled cell proliferation, immune invasion and systemic metastatic progression.^1^, ^2^ Among the panoply of malignancy, breast cancer stands out as the second most common diagnosed phenotype worldwide, reporting 1.4 million new cases reported annually. In US alone, nearly 297 790 cases of invasive BC were reported in 2023, accompanied by 56 500 non‐invasive BC cases by 2024. The sustained pervasiveness of malignancy emphasizes the need of developing next‐generation technology advancing diagnosis, molecular profiling and targeted therapeutics.^1^, ^3^, ^4^, ^5^, ^6^, ^7^

Triple‐negative breast cancer (TNBC) is clinically the most aggressive subtype of BC, lacking the presence of oestrogen, progesterone and HER2 receptors. Despite significant strides in conventional chemotherapeutics and targeted therapy, the immunosuppressive TME and therapeutic resistance cause a major challenge in its treatment. The prognosis of this BC cancer subtype is poor, with low survival rate (approx. 5 years), especially those with significant metastasis compared to other subtypes.^8^, ^9^, ^10^, ^11^, ^12^ Although some patients respond to the initial chemotherapy, changes of tumour relapse are comparatively very high due to immunosuppressive tumour microenvironment and developed chemoresistance.^9^, ^13^, ^14^, ^15^, ^16^ It frequently harbours the elevated levels of regulatory T cells, tumour‐associated macrophages and immunosuppressive cytokines, collectively dampening antitumour immune response and immune evasion.^17^, ^18^ In addition to this, the inter‐ and intratumoural heterogeneity adds more complexity in effective therapy, presenting the need for development of innovative strategies to overcome resistance, modulate TME and improve patient compliance.^17^, ^19^ In this context, immunotherapeutic strategies are being developed to catalyse innate immune responses and restore antitumour surveillance.^1^, ^20^

The stimulator of interferon genes (STING) pathway is emerging as a novel immunotherapeutic target activating production of type 1 interferons, promoting infiltration of cytotoxic T cells and maturation of dendritic cells and reprogramming innate immunity amplifying immunosurveillance. This acts as bridge between innate and adaptive immune responses sensing cytosolic DNA.^21^, ^22^ The cytosolic or mitochondrial DNA or cyclic dinucleotides trigger the STING pathway, promoting production of type 1 interferons, proinflammatory cytokines, antigen‐presenting cells and cytotoxic T cells.^23^, ^24^ In hand encouraging the maturation of dendritic cells and natural killer cells resulting, resulting in activation of significant cytotoxic immune pathways with a long‐term immunological memory for protective immune memory. Preclinical and clinical studies have evidenced that STING agonists remodel the TME by any of the above mechanism, improving therapeutic outcomes.^25^

However, the clinical translation of STING pathway modulators is constrained by poor pharmacokinetics, unintended off‐targeting and inefficient cytosolic delivery. Recent innovations in nanoparticle engineering help to overcome the challenges associated with delivery of STING agonist, offering solutions by protecting from degradation, triggering drug release and enhancing drug accumulation.^26^ A summary of major barriers for TNBC treatment is denoted in Table 1.

**TABLE 1:** Challenges and nanotechnology‐driven multimodal solutions activating stimulator of interferon genes (STING) cascade for triple‐negative breast cancer (TNBC) treatment.

Therapeutic limitation/challenge	Consequence	Multimodal solution	Example of NPs
Rapid degradation of STING agonist	Reduction in biological half‐life, induction of enzymatic hydrolysis by ENPP1‐phosphodiesterase	Encapsulation within NPs	PEI polymer layered, lipid based^68^,^67^
Entrapment of STING agonist within the endosome after cellular uptake	Inappropriate cytosolic delivery	Optimization of size sand surface zeta potential, coating with optimum endosomolytic agent	pH responsive carrier, logic gate mediated NPs^42^, ^45^, ^46^
STING‐cascade inhibition in response to hypoxia in TME	Inhibition of cGAMP‐STING agonist binding because of accumulation of hypoxia induced ADSL‐mediated fumarate	Hypoxia responsive NPs	Redox‐responsive organosilicans, azobenzene‐triggered NPs^71^, ^72^
Immune evasion and resistance	Reduction in level of T cell infiltration and upregulation of regulatory cells	Triggers immunogenic cell death	NPs inducing ferroptosis/cuproptosis, stimuli‐responsive systems^50^, ^53^
High stromal density, TME rich in fibroblast and elevated levels of interstitial pressure	Poor penetrability and targetability	Active targeting, optimization of size to enhance penetrability by EPR effect	Aptamer, folate or CD44^41^, ^69^
Non‐specific activation, triggering off targeted inflammation	Systemic toxicity	Ligand‐targeted therapy, logic gate, stimuli‐responsive	NIR‐triggered exosome‐liposomal hybrid, implantable gels^53^, ^65^
Absence of long‐term immune memory	Initial regression after tumour relapse	Co‐encapsulation of STING agonists with peptides, antigens, ligand functionalization, etc.	Nanovaccine, DMXAA‐loaded MXene^62^, ^66^
TME modulation in response to metabolic and genetic distortion	Suppression of STING transcription by MYC oncogenes	Combination therapy, epigenetic modulating NPs	PD‐L1/PD‐1 synergy, metabolism‐modulating lomitamide NPs

Abbreviations: NP, nanoparticle; PEI, polyethyleneimine.

Overall, this review aims to summarize the biological mechanism and immunological significance of the STING pathway in TNBC therapy with particular emphasis on innovative strategies used for delivering its agonists. Furthermore, it discusses the synergistic potential of combination therapy, opening new edges for its clinical translational.

### 1.1 Molecular mechanisms of STING pathway in immunomodulation and cancer treatment

Cytosolic double‐stranded DNAs, whether originating from either exogeneous sources such as viruses, parasites and intracellular bacteria or endogenous sources like mitochondrial DNA leakage or nuclear DNA damage, serve as an alarm activating the STING signalling cascade, triggering an innate immune response. This catalyses the formation of 2′3‐cGMP from cyclic GMP‐AMP synthase, activating the ER‐resident adaptor protein STING. Upon activation, STING undergoes tertramerization, oligomerization and palmitoylation within the endoplasmic‐Golgi intermediate compartment, promoting localization of TBK1 kinase. This, in turn, facilitates phosphorylation of STING at serine 366, promoting activation and nuclear translocation of IRF3 expressing type 1 interferons and stimulated genes. Simultaneously, triggering NF‐κB pathway induces proinflammatory genes and immune checkpoint marker PD‐L1 on the cell membrane.^27^, ^28^

This cascade activates a wide variety of immune cells, including cytotoxic T cells, monocytes and macrophages within the TME, facilitating tumour cell identification and disruption. Also reduce the number of regulatory T cells supporting the formation of tertiary lymphoid structure, strengthening antitumour response. In TNBC, MYC oncogenes regulating the cell proliferation and growth are either mutated or overexpressed. These oncogenes epigenetically suppress the expression of STING, helping tumour evasion from immune detection. They suppress the STING transcription by binding to the STING1 enhancer region, reducing the production of chemokines (CXCL10, CCL5, CXCL11),^29^ resulting in ‘cold’ TME due to decrease in tumour infiltration of T cells. In both primary and metastatic tumours, the overexpression of MYC is correlated with suppression of STING, contributing to resistance against immune checkpoint blockades (ICBs). Hence, by suppressing MYC oncogenes, encouraging activation of cGAS‐STING pathway may help to sensitize tumour to PD‐L1‐targeted immunotherapy.^17^ STING signalling rewires preferential activation of NF‐κB‐dependent pathway promoting cell survival and metastasis.^30^, ^31^

Mounting evidences establishes that the cGAS‐STING pathway is a promising immunotherapeutic target for cancer therapy owing to its crucial role in initiating and amplifying antitumour immunogenic response and treating immunosuppressive TNBC.^32^, ^33^ This reshapes the tumour microenvironment, promoting the maturation of dendritic cells, infiltrating the cytotoxic T cells and sensitizing TNBC to immune checkpoint blockage therapies.^34^

Literature surveys report that STING agonists in combination with chemotherapeutic agents amplify the immune responsive by activating the cascade modulating cancer‐associated fibroblast, perivascular‐like cell state, ferroptosis or cupporoptosis, suppressing the tumour growth. These findings highlight STING as an immunological hub integrating pharmacological activation with diverse drug delivery modality, enhancing spatiotemporal drug targetability.^26^, ^35^

Nanoparticle‐based delivery systems efficiently address the key challenges associated with the delivery of STING agonist therapy, including suboptimal cellular uptake, off‐targeting and rapid systemic clearance. Modified nanocarriers facilitate researchers to achieve enhanced cytosolic delivery with improved drug pharmacokinetics and robust STING pathway activation in TNBC models. These delivery platforms not only remodel the TME from immunosuppressive to immunostimulatory but also potentiate the therapeutic outcomes, offering promising strategy to improve efficacy and overcome resistance.^36^, ^37^

### 1.2 Nanoparticles‐mediated STING‐cascade activation: unveiling the immune sanctum of TNBC

Researchers had developed a diverse array of nanoplatforms to address the multifaceted barriers associated with TNBC, including immune evasion, cytosolic delivery and inactivation of metabolic pathways. These nanoconstructs are modified with logic gate, redox responsiveness, manganese bases, and so forth to facilitate the spatiotemporal drug release while maximizing immunological response and encouraging STING activation.^38^ By strategically integrating apoptotic triggers, mitochondrial DNA disruptors, cuproptosis and ferroptosis initiate immunogenic cell death, amplifying type 1 interferon and enhancing STING‐mediated immune surveillance. The subsequent section outlines the mechanistic contribution to STING‐mediated activation in TNBC.^39^, ^40^ A comprehensive overview of STING‐cascade activating NPs with their immunological outcomes is summarized in Table 2. It critically analyses the translational potential of STING pathway‐activating nanotherapeutics in TNBC.

**TABLE 2:** Mechanistic insight of stimulator of interferon genes (STING) activating NPs for triple‐negative breast cancer (TNBC) treatment.

Nanoparticle (NP)	Drug used	Cell lines	In vivo	Route	Result	Ref.
PLTs@Pt‐lipid@lomi	Miriplatin + lomitapide	4T1, DC2.4, RAW264.7	Orthotopic TNBC BALB/C mice model with post‐surgical metastasis	I.V.	Reduction in expression of cancer stem cell markers (SOX2, NANOG, OCT4) and STING‐related chemokines. A potent inhibition in tumour regrowth and lung metastasis was observed	^[^ ^44^ ^]^
Fe^0^ @HMON@DNA‐Ex	For exosome priming SN38	4T1 (TNBC), DCs, RAW 264.7	Orthotopic and metastatic TNBC (BALB/C)	I.V.	Selective Fe^0^ release in response to GSH/pH present in TME results in activation of ferroptosis and STING pathway. Results in prevention of metastasis, tumour suppression and DC maturation	^[^ ^46^ ^]^
HA‐SS‐CPT@Ce6 NPs	Camptothecin + chlorin e6	4T1 (TNBC)	Orthotopic and metastatic TNBC (BALB/C)	I.V.	An enhancement in cellular internalization was observed in response to CD44 receptors, followed by GSH‐triggered camptothecin release in response to laser‐induced ROS production in hypoxic condition. A significant downregulation of Bcl‐2 and elevation of cleaved caspases was observed, initiating STING pathway, and suppression of tumour growth was observed. The histopathological examination of 4T1 mouse tissues augmented tumour necrosis while preserving structural integrity of organs	^[^ ^63^ ^]^
GelMA‐CMO Xerogel Puncture Implant	Cu_0.5_Mn_2.5_O_4_ (CMO) NPs	4T1 (TNBC), bone marrow‐derived dendritic cells (BMDCs)	Re‐section of 4T1 orthotopic breast cancer model and metastatic TNBC (BALB/C mice)	Local implantation	ROS generation, induction of cell cuproptosis and cGAS‐STING pathway provoke immunogenic cell death facilitating maturation of dendritic cell and amplification of immune response. Local tumour recurrence and lung metastasis	^[^ ^53^ ^]^
FA‐IR780/EGCG@MnO2	EGCG, IR780, Mn^2+^	4T1 (TNBC), bone marrow‐derived dendritic cells (BMDCs)	4T1 orthotopic TNBC BALB/C mice model and B16F10 melanoma model	I.V.	GSH triggered release of EGCG, IR780, Mn^2+^ with initiation of STING cascade and Fenton reaction. The oxidative stress leads to mitochondrial ROS production, lysosomal escape and mtDNA leakage. Primary tumour inhibition up to 84.9% was observed with no systemic side effects	
Double‐layer PEI‐coated CPGP	cGAMP	RAW 264.7, CT26	4T1 orthotopic breast cancer model, CT26 BaLB/C mice	I.V.	Combination of αPD‐1 with CPGP demonstrated significant tumour suppression 96.3% compared to free cGAMP. Along with this, more pronounced histological damage was observed compared to other cohorts. In line with the above results, a marked increase in cytokinin levels of approx. 2.7‐fold over CP group was observed activating STING pathway leading to maturation of dendritic cells	^[^ ^45^ ^]^
AMFL (exosome–liposome hybrid NP)	TBTP‐Bz + MSA‐2	4T1 (TNBC)	Bilateral, 4T1 tumour bearing mice (TNBC)	I.V.	Induction of immunogenic cell death suppressed primary and metastatic tumours. The disruption of exosome–liposome hybrid NP in response to laser irradiation of 808 nm leading to 90% cell necrosis Histopathological interpretation of data demonstrated no sign of toxicity in heart, spleen, lung and kidney	^[^ ^65^ ^]^
Ce6/PTX‐loaded cRGD lipid‐modified nanobubble	Paclitaxel (PTX) + chlorin e6 (Ce6)	4T1 (murine breast cancer)	4T1 orthotopic breast cancer model and lung metastatic model	I.V.	Significant metastatic inhibition up to 93.9%, enhanced CD8+ and T cell infiltration, activating STING pathway synergising with PD‐L1 bloker	^[^ ^66^ ^]^
Co_2_ polymeric nanoparticle encapsulating cobalt(III) cyclam‐based prodrug	Cobalt(III) cyclam‐based prodrug + STING agonist MSA‐2	MCF‐7, MDA‐MB‐231, 4T1 (murine breast cancer),	BaLB/C mice bearing 4T1 orthotopic breast cancer model, bilateral and lung metastatic model	I.V.	57% of tumour inhibition was observed in 4T1 murine mice model surpassing cisplatin efficacy (36%), activation of STING pathway leading to infiltration of IFN‐β, IL‐6 and IL‐12 in response to phosphorylation of TBK1 and IRF3. Prolonged survival and nearly completed tumour irradiation on combination with anti‐PD‐1 blocker	^[^ ^64^ ^]^
Ti3C2@Au‐PEG‐DMXAA	DMXAA	4T1, RAW 264.7	4T1 orthotopic tumour bearing BALB/C mice	Intratumoural	Strong immune activation, long‐term memory and nearly complete irradiation of tumour. TAPD‐mediated mPTT‐induced immunogenic cell death was confirmed by in vitro assay. Along with activation of cGAS‐STING–cascade, the phosphorylation of TBK1, IRF3 and STING surged Microscopic evaluation confirmed pronounced tumour necrosis with minimal off‐target organ damage maintaining normal histopathology	^[^ ^62^ ^]^
Mn/Se‐Gem nanosheets	Selenium + gemcitabine	RAW 264.7 cells	4T1 orthotopic tumour bearing BALB/C mice	Peritumoural (S.C.)	80% tumour inhibition with enhanced cellular apoptosis and reduction of M2 macrophages	^[^ ^59^ ^]^
OHA@Lys/PHL Mn^2+^	Phloretin (PHL)	4T1 (murine breast cancer)	4T1 orthotopic tumour bearing BALB/C mice	Intraperitoneal	46.4% tumour inhibition on monotherapy and increase up to 86.7% with combination therapy with phloretin and Mn^2+^. this activates the cGAS‐STING cascade surging cell necrosis and reduction in metastasis	^[^ ^58^ ^]^
Manganese dioxide‐doped mesoporous organosilica nanoparticles (MMONs)	–	4T1 (murine breast cancer), PAN02, Hepa 1–6, MDA‐MB‐231	4T1 orthotopic tumour bearing BALB/C mice	S.C.	TPP‐MMONs possessed a positive zeta potential of +4.92 mV and a uniform diameter of 250 nm confirming their colloidal stability It exhibited a potent tumouricidal activity	^[^ ^61^ ^]^
CZP NPs	Anti‐PD‐L1 antibody + phothermal irradiation	4T1 murine TNBC	4T1 orthotopic breast cancer BALB/C mice	Inratumoural, intraperitonial	>85% reduction in tumour volume and lung metastasis favouring increase in M1/M2 macrophage ratio, nearly 93.8% cell death with NIR Strong cuproptosis and activation of cCAS‐STING cascade. Nanoparticle was approximately 177.2 nm in size	^[^ ^50^ ^]^
Mn@SCD1i@αPD‐L1	Stearoyl‐CoA desaturase 1 + anti‐PD‐L1 antibody	4T1 murine TNBC	4T1 orthotopic breast cancer BALB/C mice	Inratumoural, intraperitonial	Mn@SCD1i@αPD‐L1 group had 26.2% rate of maturation of dendritic cell indicating activation of cCAS‐STING pathway positioning the nanoparticle (average hydrodynamic diameter ∼120 nm) as promising strategy for treatment of immunosuppressive TNBC An was observed	^[^ ^47^ ^]^

Abbreviation: CPGP, covalent organic polymer; mtDNA, mitochondrial DNA.

### 1.3 Biomimetic nanoparticle for STING‐cascade‐mediated activation and synergistic PD‐1/PD‐L1 blockades

Metastasis remains the predominant biological escape hack leading to mortality in TNBC patients. The process is initiated by the dissociation of circulatory tumour cell (CTC) from the primary tumour, followed by platelet aggregation and dissemination by lymphatic and haematogenous pathways. These cells easily impair immune visibility and colonize at distinct site. Conventional therapies often fail to counter these migrating cells, emphasizing the critical need of novel therapeutics.

Ongoing advancements in nanotechnology have enabled the fabrication of biomimetic nanoparticles to deliver STING agonists directly to static and distinct metastatic tumour cells, thereby activating innate immunological pathways, suppressing immune evasion and enhancing the cytotoxicity. They convert immunologically ‘cold’ tumours into ‘hot’ ones by increasing, thereby increasing their responsiveness to immunotherapy.^41^, ^42^ STING agonist initiates an innate and adaptive immune response via triggering the pathway leading to production of type 1 interferons and other proinflammatory cytokines, promoting maturation of dendritic cells and activation of T cells.

In addition to this, the activation of STING cascade upregulates the expression of PD‐L1 enhancing the efficacy of ICB therapies. This synergy plays a critical role in modulating the immunosuppressive TME of TNBC, favouring the co‐administration of PD‐L1 inhibitors with STING‐activating nanoparticles.^43^

The major obstruction associated with the delivery of STING is that it resides within the cell cytosol. To overcome this intracellular delivery of cyclic dinucleotides, STING agonist is required. Cheng et al. addressed this by designing a liposomal nanoparticle to deliver cyclic guanosine monophosphate‐adenosine monophosphate (cGAMP), amplifying innate immune response. It upregulates the macrophages—MHC‐I, MHC‐II and CD 86—facilitating presentation of antigen and T cell priming. The in vivo studies demonstrated suppression of tumour growth and immune evasion in TNBC model, favouring the rationale of using PD‐L1 inhibitors within STING‐activated nanocarriers. The formulation achieved high encapsulation efficiency (∼43%) with optimum particle size (∼85 nm) and zeta potential (∼14.8 mV), enabling I.V. administration for systemic delivery.^42^

The cGAS‐STING pathway is rapidly emerging as a key for the treatment of solid tumour more specifically breast cancer. Despite its promising therapeutic potential, clinical utility remains constrained by low biostability, inefficient tumour localization and off‐target tumour delivery.

To overcome these limitations, an approach to target single CTCs instead of CTC clusters is favoured. In this response, a biomimetic nanoplatform was designed by Fan et al. in order to navigate this hurdle. Lomitapide, a microsomal triglyceride transfer protein inhibitor, was encapsulated within a metal complex lipid‐based nanoparticle. Pt‐lipid@lomi NPs featured a core of miriplatin encapsulated within the platelet membranes, enhancing rigidity and deeper penetration. This not only enhances the cholesterol‐modulating efficacy of lomitapide but also activates the STING pathway by encouragement on cancer cells by platelet membranes.

**FIGURE 1:**
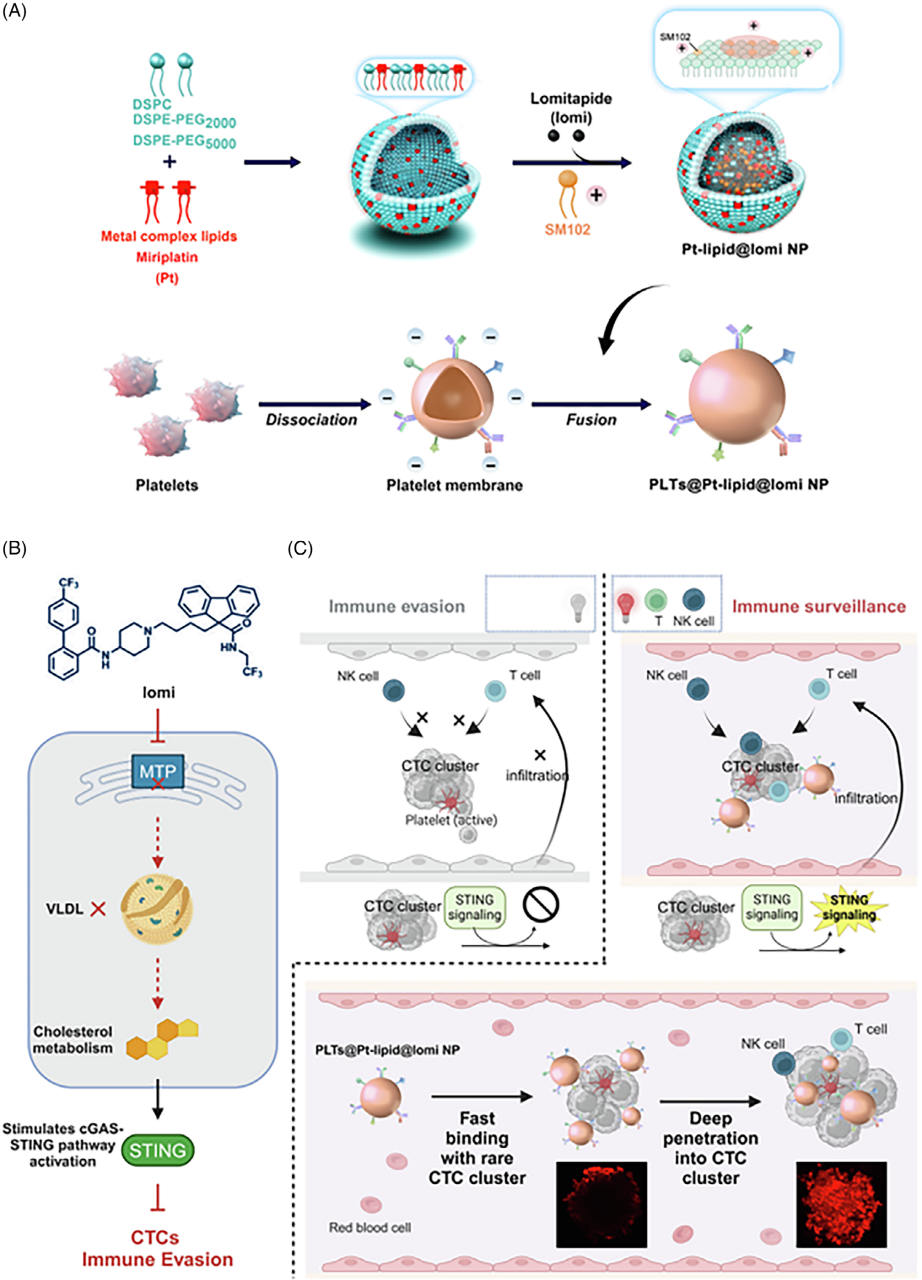
Design features and mechanism of action of PLTs@Pt‐lipid@lomi NPs (by Figdraw and Biorender). (A) Core Pt‐lipid@lomi NPs containing lomi consist of DSPC, PEGylated phospholipids (DSPE‐PEG) and miriplatin, forming metal lipid complexes. These are assembled with positively charged SM102 to form the NP's shell, which is then enveloped in a platelet membrane. (B) Lomi mechanism of action. As an microsomal triglyceride transfer protein (MTP) inhibitor, lomi regulates cholesterol metabolism, activating the cGAS‐stimulator of interferon genes (STING) signalling pathway and inhibiting immune evasion by circulatory tumour cells (CTCs). (C) The inherent rigid structure of NPs prolongs their circulation in the bloodstream, rapidly binds to CTC clusters within the blood vessel and enhances penetration into CTC clusters, thereby recruiting T cells and NK cells to infiltrate into CTC clusters and inhibiting immune evasion from CTC clusters.^44^.

A downregulation in cancer stem cell markers (SOX2, NANOG and OCT4) and STING‐related chemokines was observed in a post‐surgery animal model. A consistent body weight was observed with prolonged survival extended over 100 days, indicating no apparent adverse effect. The formulation significantly suppresses pulmonary metastasis and tumour regrowth, representing a promise for next‐generation immunotherapies (Figure 1).^44^

Building upon the need to tackle the hurdles associated with the physicochemical barriers in STING delivery, Liang et al. designed TME‐responsive nanoparticle facilitating the cytosolic delivery of negatively charged cGAMP. This molecule is generated in response to enzymatic conversion of adenosine triphosphate and guanosine triphosphate into a potent STING agonist 2′3′‐cGAMP.

This was loaded within a polyethyleneimine (PEI)‐modified covalent organic polymer (CPGP) to enhance intracellular delivery. An additional layer of PEI was coated to the above exhibiting a reduction in zeta potential of final formulation, which helped to avoid premature drug leakage and enhancement of stability promoting endosomal escape. The pH‐dependent drug release profile with a significant rise to 78.7% at pH 5.5 showcases the structural rigidly during circulation.

**FIGURE 2:**
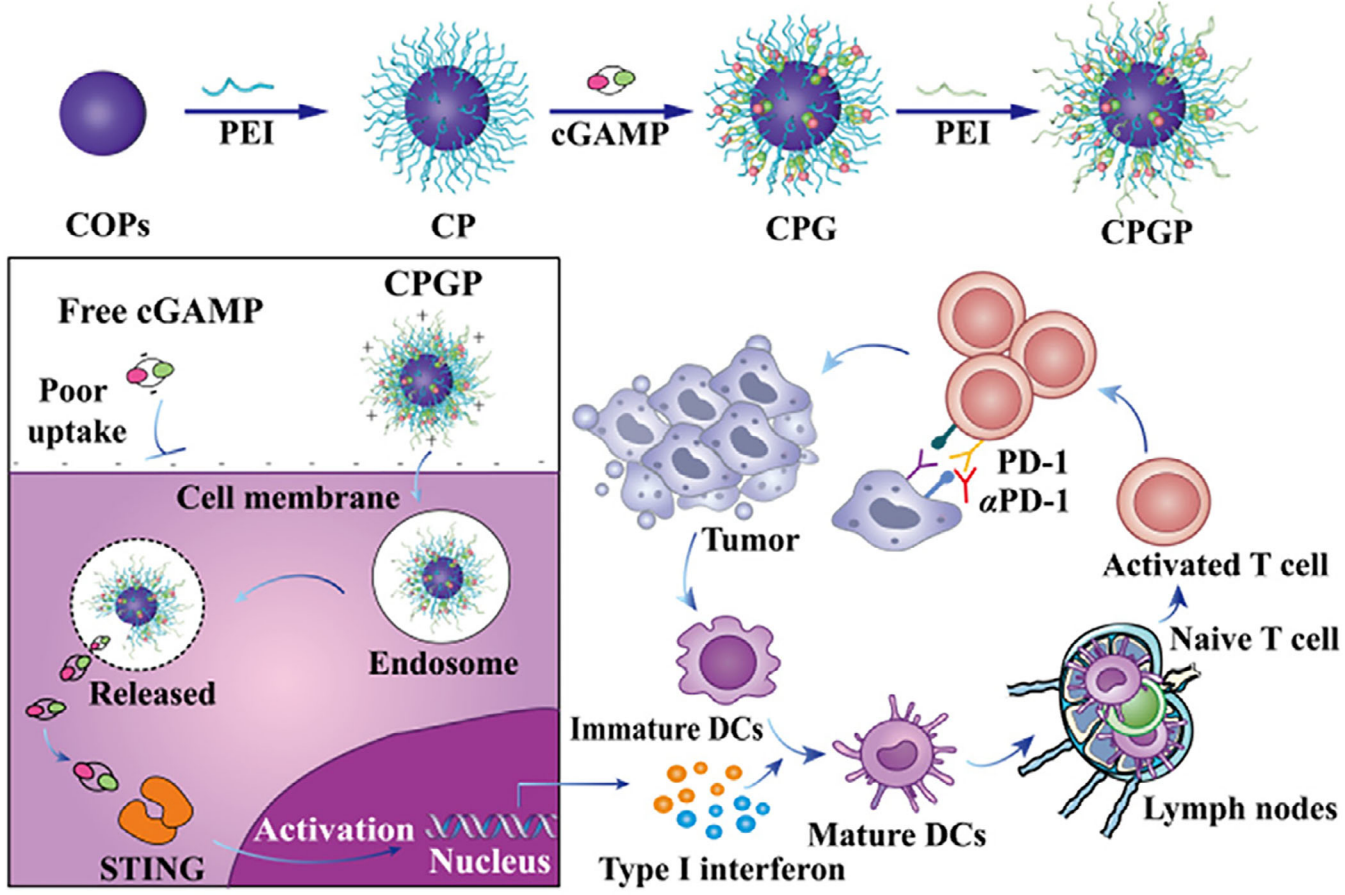
Schematic overview of the fabrication of covalent organic polymer (CPGP) and its mechanism for stimulator of interferon genes (STING) activation and immune responses.^45^

The double‐layered PEI structure of CPGP significantly enhanced cellular uptake by 5.9 folds compared to CPG, likely due to positively charged surface (24.6 mV) which enable efficient intracellular interaction with cellular membrane. This translated into increased therapeutic efficacy in vivo. Compared to PBS control group, free cGAMP and single‐layered CPNPs demonstrated modest tumour suppression, whereas a significant suppression marked up to 84.9% was observed in CPGP. This 2.3‐fold increment was more pronounced on addition of αPD‐1 (96.3%) showcasing a synergistic effect. Overall, the study presents a versatile platform overcoming the physicochemical barriers associated with the delivery of nano‐STING agonists (Figure 2).^45^

### 1.4 Dual activation of STING cascade by Trojan horse logic gate and ferroptosis in TNBC

The therapeutic constraint in treatment of TNBC is not just the absence of receptors but its systemic resistance to immune cell activation and ferroptotic vulnerability.^35^ To decode this, Guo et al. designed a concept of biological logic gates that unravel the complexities of tumour microenvironment and strategically transform the cancer therapy. DNA fragments containing exosomes derived from SN38‐treated 4T1 cells and coated mesoporous organosilica nanoparticles (MMONs) were designed to simultaneously activate STING pathway and ferroptosis. The AND gate triggered ferroptosis in response to low pH and high levels of glutathione within the TNBC cells, whereas OR gate recognized the DNA fragments and activated STING cascade in antigen‐presenting cells. Mechanistically the release of Fe^0^ initiates the Fenton reaction cascade, producing reactive oxygen species, mitochondrial dysfunction and lipid peroxidation, validating the upregulation of IFN‐α and IFN‐β. The change in intracellular levels of ROS, LPO, GPX4, ACSL4 and Fe^2+^ with significant suppression of tumour growth in TNBC models demonstrated induction of ferroptosis and activation of STING pathway within the TME. The formulation suppressed tumour proliferation, exaggerating the potential of exosomal DNA as a personalized immunogenic fingerprint for designing of nanovaccine.^46^

**FIGURE 3:**
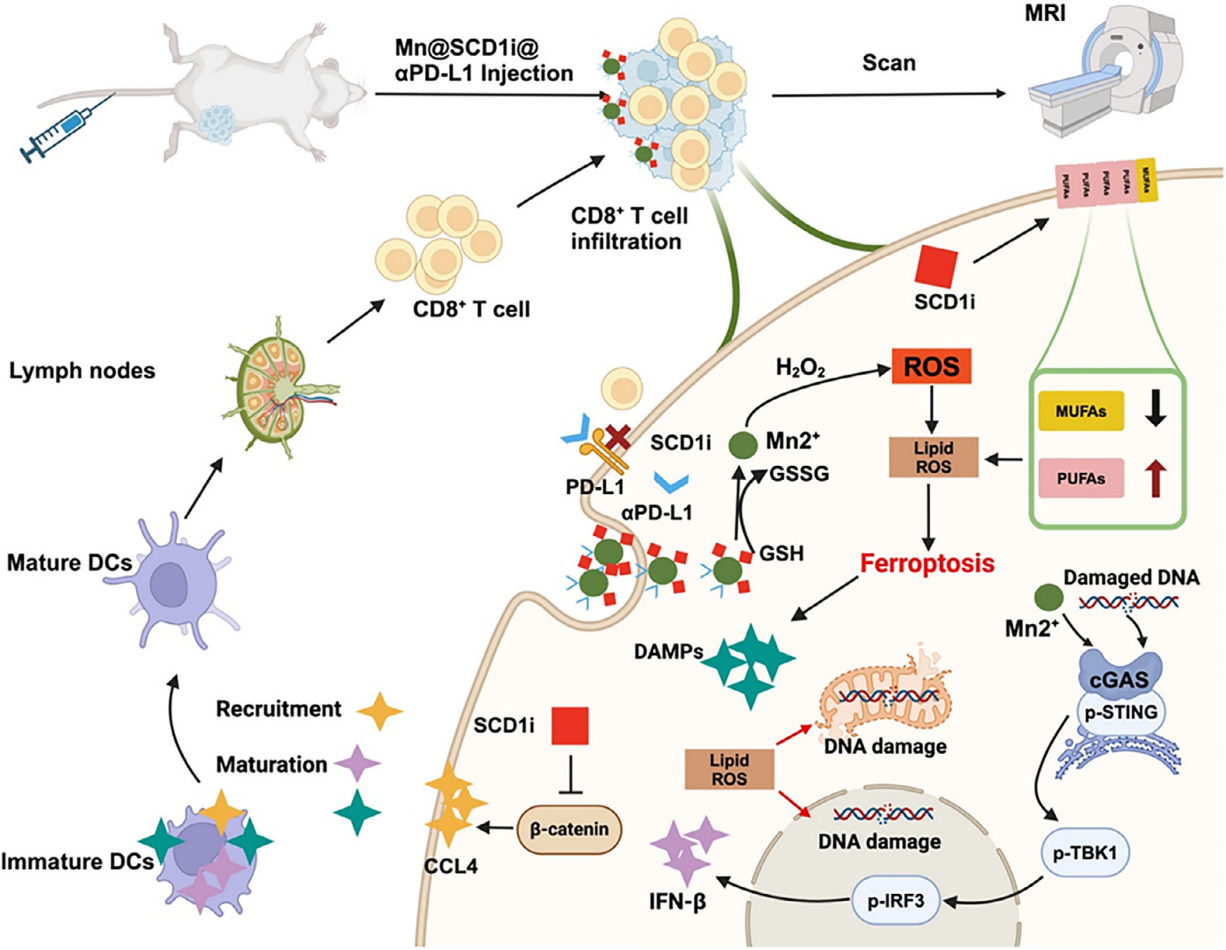
Schematic diagram for therapeutic mechanism of Mn@SCD1i@αPD‐L1. Mn@SCD1i@αPD‐L1 induces ferroptosis storm by lipid acid metabolism reprog ramming, achieving metal‐immunotherapy and monitors the treatment by MRI real‐time imaging.^47^

Ferroptosis is an iron‐catalysed immunogenic response triggering release of damage‐associated molecular patterns, causing cell death in TNBC cells by rupturing DNA. This causes maturation of dendritic cells, activating cCAS‐STING pathway. But the presence of stearoyl‐CoA desaturase 1 (SCD1) in altered TME suppresses ferroptosis. To address this limitation, Zhou et al. designed GSH‐responsive Mn_3_O_4_ nanoparticles linking SCDI inhibitor and αPD‐L1, an immune checkpoint inhibitor. Mn@SCD1i@αPD‐L1 facilitates the release of Mn^2+^, SCD1i and αPD‐L1 through glutathione depletion within the TME. Along with this, SCD1i suppresses synthesis of MUFA and sensitize ferroptosis by incorporation of PUFA in cell membrane phospholipid. A suppression in Wnt/β‐catenin pathway was observed up to 39% in both Mn@SCD1i and SCD1i groups compared to PBS group, facilitating CCL4‐mediated dendritic cell recruitment. In vivo studies demonstrated that Mn@SCD1i@αPD‐L1 group had 26.2% rate of maturation of dendritic cells, indicating activation of cCAS‐STING pathway, positioning the nanoparticle as promising strategy for treatment of immunosuppressive TNBC (Figure 3).^42^, ^47^

In addition to these, other studies developed bimetallic peroxides, ruthenium‐based nanoparticles and dual‐ion nanostimulators for simultaneous activation of ferroptosis and the cCAS‐STING pathway. Apoferritin nanoparticles were encapsulated within an arene binuclear ruthenium(II) complex to enhance mitochondrial accumulation in TNBC cells. This led to mitochondrial damage‐induced ferroptosis with subsequent activation of cCAS‐STING cascade, minimizing tumour evasion and systemic toxicity.^48^, ^49^ Polydopamine‐coated zinc‐copper nanoparticles and bimetallic peroxides containing zinc‐copper release Zn^2+^ and Cu^2+^ ions within the TME. They induce Fenton‐like reaction, generating hydroxyl ions, amplifying oxidative stress and immunogenic cell death and enhancing adaptive immunity. In combination with PD‐L1, it significantly suppressed metastasis and tumour growth in TNBC models.^47^, ^50^ Although dual‐ion hybrid nanostimulators (Ca and Mn) induce ferroptosis by glutathione depletion, mitochondrial Ca overload and ROS generation. This activates the STING pathway promoting production of dendritic cells and infiltration of cytotoxic T cell.^51^, ^52^ Hence, the coordinated induction of these approaches presents a robust strategy for treatment of TNBC.

### 1.5 Activation of STING cascade by cuproptosis

In the compelling advancements for treatment of TNBC, photothermal amplified cuproptosis has emerged as a promising therapeutic modality. Unlike other mechanisms of mitochondrial disruption (ferroptosis or apoptosis), cuproptosis destabilizes iron‐sulphur clusters, aggregating lipolytic proteins. This leads to development of mitochondrial stress, releasing DNA and activating STING cascade for immunogenic response, converting cold tumour into hot one.

TNBC, often referred to as cold tumour due to its immunosuppressive nature, poses changes in treatment. The vascular basement significantly obstructs the effectiveness of nanoparticle‐based immunotherapy. A study by Jiang et al. effectively bridges the gap of post‐surgical oncological research by modulating the oxidative stress and immune activation by cGAS‐STING pathway. The study engineered a biodegradable tumour implant containing a photo‐cross‐linked methacrylated gelatin (GelMA) xerogel, deliverable via a puncture needle upholding a sustained in vivo drug release for a period of 15 days. Triggered by enzyme matrix metalloproteinase, the implant facilitates a persistent release of Cu_0.5_Mn_2.5_O_4_ (CMO) NPs and monomethyl fumarate, promoting liberation of mitochondrial DNA. A sequential induction of cell cuproptosis and cGAS‐STING pathway provokes immunogenic cell death facilitating maturation of dendritic cell and amplification of immune response.

Cell cycle analysis brought into focus that the treatment with CMO NPs led to marked infiltration of 4T1 cells in the early G1 phase with a sharp reduction in G2/M phase. Further cell apoptosis flow cytometry assay presents a marked increase in early and late apoptosis rate reaching up to 14.81%. These findings suggest that implant triggered cellular membrane disruption, inducing cell necrosis or apoptosis. A marked reduction in local tumour reoccurrence and distinct metastasis was observed after post‐re‐section of 4T1 orthotopic breast cancer model suggesting enhancement in immunotherapeutic sensitivity of breast cancer to immune checkpoint inhibitors. Building on these findings, the study opens a new array for breast cancer treatment by seamlessly integrating redox‐responsiveness, biomaterial engineering and immune activation by cGAS‐STING pathway.^53^

Extending upon this framework, Zhou et al. developed polydopamine‐coated zinc‐copper bimetallic nanoplatforms (CZP NPs) that integrate photothermal therapy with induction of cuproptosis by copper‐zinc peroxide metal ions activating the cGAS‐STING cascade. On exposure to near‐infrared irradiation, the CZP NPs induce a local hyperthermia liberating Zn^2+^ ions, Cu^2+^ and hydrogen peroxide in acidic TME. These components synergistically trigger production of cytotoxic hydroxyl radicals in response to Fenton‐like reaction‐generated oxidative stress. This leads to disruption of mitochondrial DNA (mtDNA) causing clustering of Fe‐S proteins and lipolytic protein aggregation. Simultaneously, zinc ions help to enhance the catalytic activity of cGAS causing phase separation, amplification of type 1 interferon signalling and PD‐L1 upregulation, initiating a strong immunogenic response.

Physicochemical characterization revealed that ZnO_2_ and Cz nanoconstructs exhibited a zeta potential of 37.0 ± 0.42 and 29.7 ± 0.78 mV, respectively, which eventually dropped down to −21.2 ± 1.13 mV by surface modification with polydopamine confirming successful modification. CZP nanoparticles exhibited rapid 4T1 cell internalization provoking concentration‐dependent cell death upto 98.1% upon photothermal activation using NIR at 40 µg/mL concentration. The formulation displays remarkable stability and photoconversion efficiency (*ŋ* = 22.29%) across repeated thermal cycles.

**FIGURE 4:**
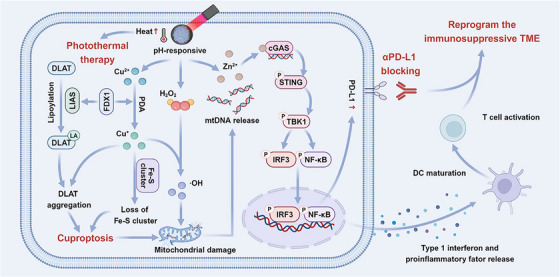
Schematic diagram of the Cu‐ZnO2@PDA nanoplatforms combined with αPD‐L1 for enhancing triple‐negative breast cancer (TNBC) immunotherapy.^50^

Furthermore, the combination of CZP with anti‐PD‐L1 therapy with NIR irradiation enhanced infiltration of cytotoxic CD^8+^ and T cells suppressing regulatory T cells. In vivo assessment demonstrated >85% reduction in tumour volume and lung metastasis favouring and increase in M1/M2 macrophage ratio. Altogether the study uncovers a promising avenue for treatment of immunosuppressive tumours, especially TNBC (Figure 4).^50^

### 1.6 Manganese‐based nanoparticles amplifying mitochondrial‐driven immune response and STING cascade in TNBC

Manganese‐based NPs are gaining prominence as a robust platform enhancing the immunotherapeutic efficacy of loaded agent in TNBC intensifying mitochondrial‐driven immune response along with activation of STING cascade. These NPs provoke the release of mitochondrial DNA into the cytoplasm promoting the infiltration of type 1 interferons and production of reactive oxygen species.^54^ Concurrently, promoting immunogenic cell death driving STING cascade enables enhancement in antitumour activity. Advanced Mn‐NPs, including manganese‐phenolic network, hybrid nanocomposite and manganese‐dioxide based, are tailored for TNBC therapy.^55^, ^56^, ^57^

Pushing forward the combination of immunotherapy and chemotherapy, Wang et al. engineered a cutting‐edge therapeutic approach co‐loading phloretin and manganese‐coordinated oxidized hyaluronic acid within a nanoparticle. OHA@Lys/PHL Mn^2+^ designed nanoconstruct was conjugated with oxidized hyaluronic acid, forming Schiff base linkage, enabling CD44 receptor targeting and activation of cGAS‐STING pathway in response to Mn^2+^ ions. The monodispersed nanoparticles have a mean diameter of approx. 115 nm demonstrating excellent pharmacokinetic and colloidal stability. Mechanistically the formulation triggers production of mitochondrial ROS, expressing γH2AX (a biomarker of DNA damage) and elevated Bax/Bcl‐2 ratio, indicating activation of apoptotic pathway. Immunophenotyping analysis indicated that OHA@Lys/PHL Mn^2+^ potentiates maturation of dendritic cells up to 48.5% in 4T1 tumour bearing mice compared to 28.2% in control group. In vivo studies demonstrated that monotherapy suppressed tumour growth by 46.4% with a marked increase up to 86.7% with a statistical reduction in lung metastasis. Building up to these findings, histopathological analysis revealed a marked reduction in Ki67 proliferation index and extensive cell necrosis with no major distortion of organ morphology. Collectively, these findings offer an approach for clinical translation of chemo‐immunotherapeutic modalities.^58^

Adding more to this, Zhang et al. structured pH sensitive manganese nanosheets co‐loaded with nano‐selenium and gemcitabine. Hyaluronic acid‐modified selenium nanoparticles incorporated in nanosheets enhanced therapeutic efficacy of gemcitabine with minimal systemic toxicity and enhanced targetability. The quasi‐hexagonal morphology of nanosheets with an average size of <200 nm and zeta potential of −14.4 mV visualized sustained dispersion and enhanced colloidal stability. In vitro study demonstrated potent cytotoxicity in 4T1 cell line with reduction in cell viability to 20% at 25 µM. Approx. two‐fold increase in ROS levels resulting in mitochondrial membrane depolarization and apoptosis was observed in Mn/Se‐Gem compared to controlled group. TUNEL and H&E staining revealed approx. 47.4% early apoptosis and total apoptosis of 63.7% within tumours tissue. Sequential in vivo analysis indicated 80% tumour inhibition in 4T1‐bearing mouse model surpassing monotherapy of MnO (˜35%) and gemcitabine (˜56%). Collectively, the nanosheets bridge clinical translatability with chemotherapeutic precision with targeted immune potentiation leveraging STING activation. By leveraging selenium‐based ROS modulation, Mn/Se‐Gem nanosheets present a blueprint for oncology.^59^

**FIGURE 5:**
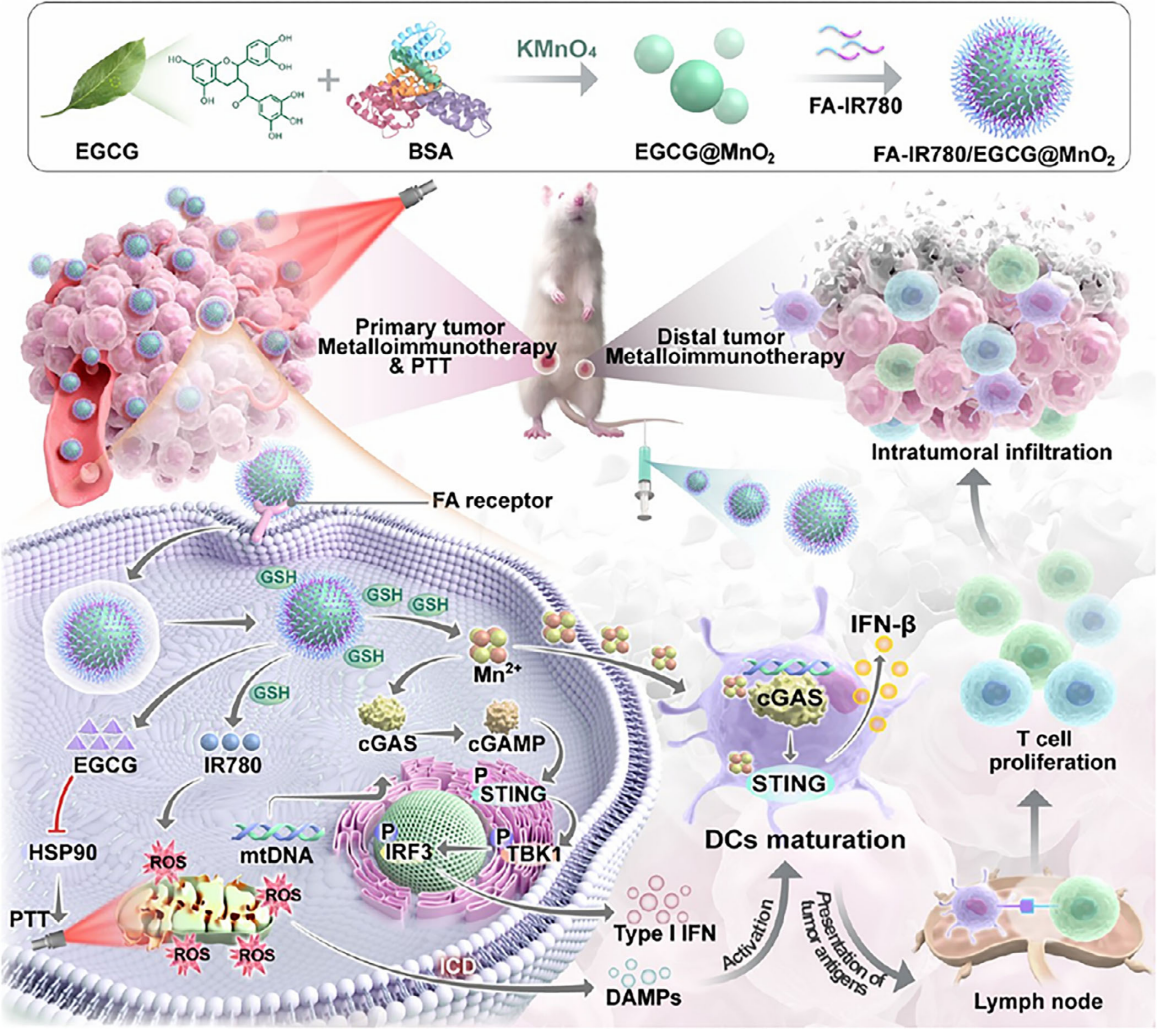
Preparation of the nanoagonist (FA‐IR780/EGCG@MnO_2_ article) and the mechanism of the nanoagonist inducing immune response against triple‐negative breast cancer (TNBC).^60^

Building on these findings, Mu et al. designed a strategy to integrate photothermal therapy with a cascade amplifying immune agonist to prepare a manganese‐based nanoagonist (FA‐IR780/EGCG@MnO2). The study specifically targets TNBC and melanoma by generation of mitochondria‐targeted ROS inducing immunogenic cell death by releases mitochondrial DNA. Metalloimmunotherapy helps to harness the immunomodulatory potential of metal ions enhancing antitumour efficacy by secretion of IFN‐1, IL‐6, TNF‐α and IL‐8 in multidrug resistant patient. The nanoparticle was prepared by a multiple‐step process initiated by production of MnO_2_ nanoparticles through л–л stacking interaction and oxidative coupling. The process was completed via a redox reaction between EGCG and KMnO_4_ and surface modification with a near‐infrared photothermal dye IR780. To enhance targetability, folic acid was conjugated to the surface by PEG linkers. Upon 4T1 cellular internalization, the nanoparticles efficiently escape lysosome and release nanoagonist in response to the elevated level of glutathione and low pH. The released Mn^2+^ triggers Fenton‐like reaction, yielding OH radicals, intensifying oxidative stress and amplification of STING pathway. In vitro drug release of thermally responsive nanoagonist validates STING pathway activation, endosomal escape and strengthened antigen presentation. In line with the above results, 4T1 orthotopic TNBC model and B16F10 melanoma model presented a suppression in tumour growth and metastasis. Histopathological examination further revealed tumour necrosis, reduction in Ki‐67 proliferation and preservation of organ integrity validating dual outcomes of safety and efficacy. The findings underscore the synergistic potential of STING‐cascade activation and metalloimmunotherapy transforming innate immune response and TME (Figure 5).^60^

As discussed in previous studies, cGAS‐STING pathway provokes immunogenic response in TNBC. Still, the conventional method of nuclear activation is limited to the multiple protective nuclear barriers and histone proteins enveloping the cytosolic DNA limiting its detection. In contrast, mitochondrial DNA is more prone to oxidative stress and cytosolic leakage, making it a more pronounced and feasible trigger for STING activation. This highlights the pursuit of strategies harnessing disruption of mtDNA for enhancing innate immunity. In light of Zhong et al. introduced, a redox‐responsive MMON doped with manganese dioxide. They were further functionalized with triphenyl‐phosphine (TPP) a mitochondrial‐targeting unit, yielding TPP‐MMONs to enhance GSH‐responsive mitochondrial toxicity by releasing Mn^b+^ ion TME. This induces mitochondrial damage facilitating initiation of cGAS‐STING cascade in response to mtDNA. Experimental data of in vivo and in vivo assays affirm that TPP‐functionalized MMONs stimulate immunosuppressive TME of TNBC promoting maturation of dendritic cells and infiltration of associated biomarkers. In combination with αPD‐L1, a significant tumour suppression was observed in distant and metastatic lesion prolonging survival of 4T1 tumour bearing mice model. The GSH responsiveness of TPP‐MMONs was confirmed by observing the structural distortion into a bowl‐shapes structure by TEM imaging interpreting the breakdown of Mn–O bonds. Additionally, the structural rigidity of nanoparticles was maintained with negligible release of Mn under acidic conditions (pH 5.5), simulating the lysosomal environment and confirming their ability to traverse to the mitochondria.

**FIGURE 6:**
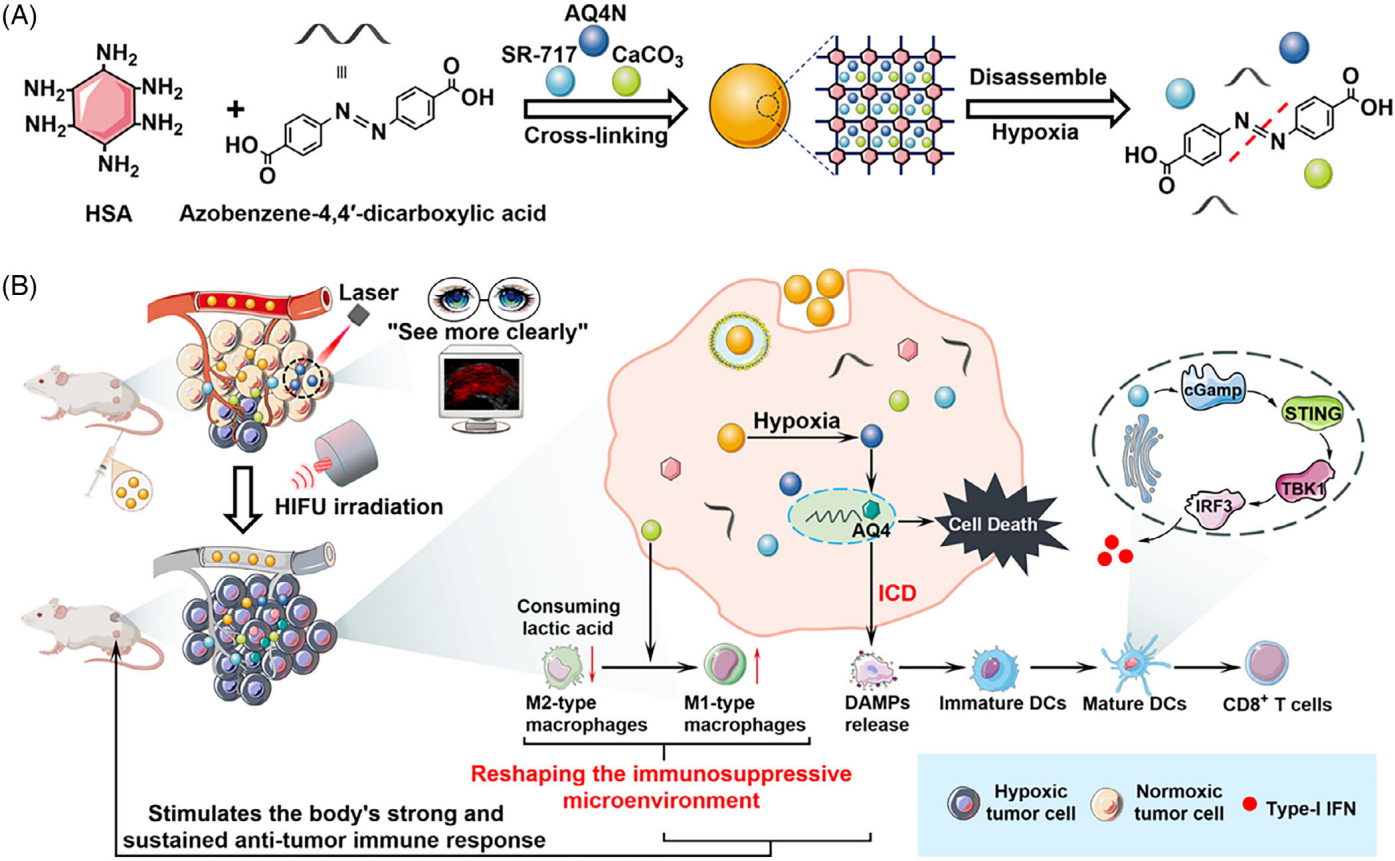
Preparation and mechanisms of action of HACS NPs. (A) Cross‐linked HACS NPs preparation process. (B) Schematic illustration of enabling PA imaging‐guided HIFU surgery via HACS NPs and their mechanisms of eliciting antitumour immunity to suppress primary/distant tumours by improving ICD and activating the stimulator of interferon genes (STING) pathway.^63^

**FIGURE 7:**
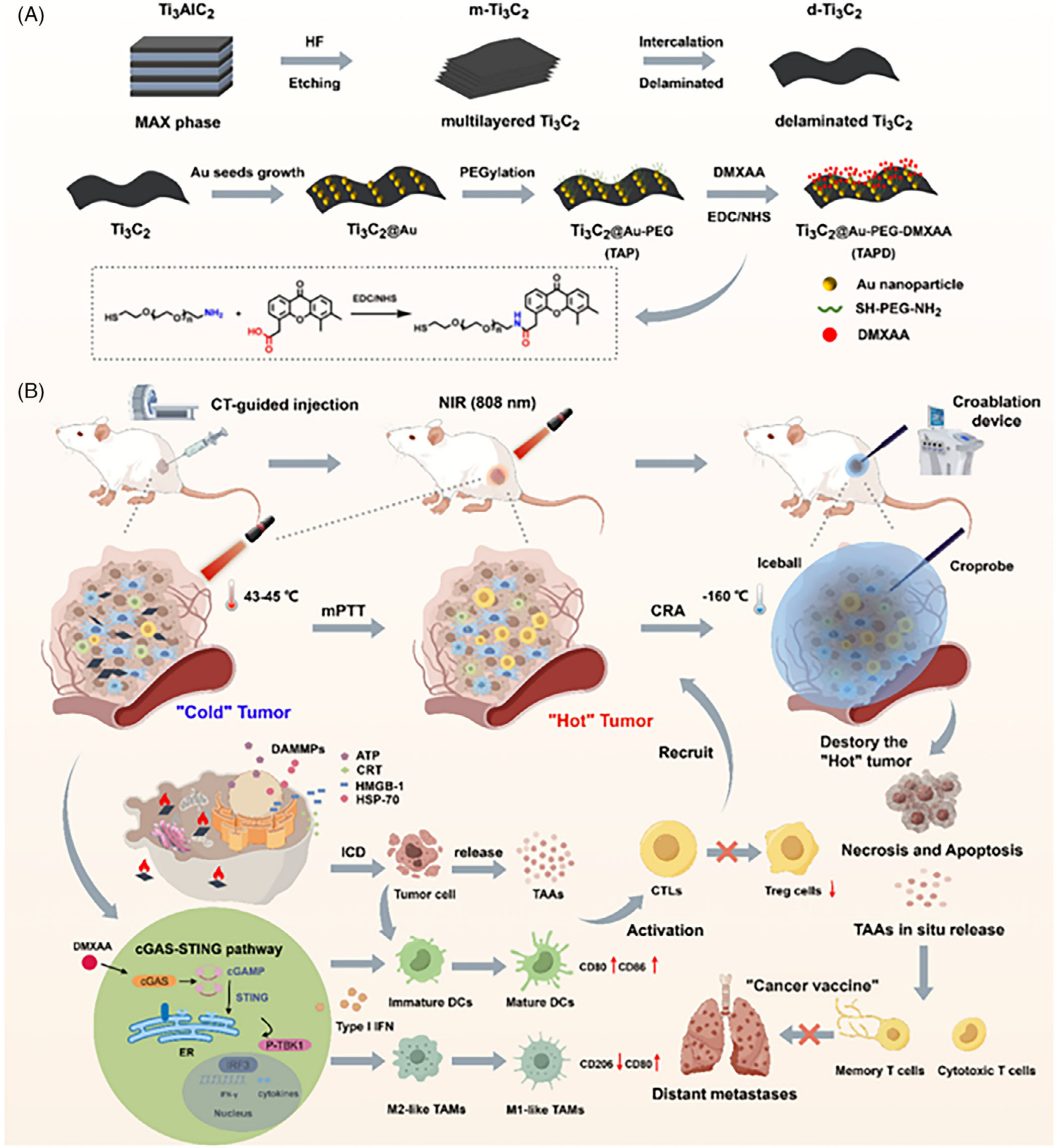
A schematic illustration of the preparation of Ti3C by Figdraw. Ti3C2 @Au‐PEG‐DMXAA (TAPD) functional nanosheets and the synergistic tumour therapeutic modality.^62^

The cytotoxicity assay against 4T1 cells marked a significant rise in cytotoxicity with TPP‐MMONs showing 1.57‐fold lower IC_50_ value compared to MMONs. Additionally, upregulation in PD‐L1 expression by 1.4‐fold over non‐targeted nanoparticles indicated enhanced immunomodulation (Figures 6 and 7).^61^, ^62^

### 1.7 Multimodal nanoparticle activating STING cascade in TNBC: strengthening tumour‐specific immune response

One of the major obstacles in treatment of solid tumour is systemic toxicity and spatiotemporal drug release. Traditional chemotherapeutic agents preferentially demonstrate a lack of accumulation in TME. High‐intensity focused ultrasound holds a significant potential for treatment of non‐invasive breast cancer, but its application is restrained by hypoxia responsive immunosuppressive TME. To address this, Huang et al. combined chemotherapy with photodynamic therapy integrating topoisomerase I inhibitor camptothecin with chlorin e6 a photosensitizer within albumin‐based nanoparticles.

A combination of hypoxia‐activated prodrug AQ4N, CaCO_3_ and STING agonist (SR‐717) was encapsulated within albumin cross‐linked nanoparticles. A marked enhancement in release rate up to 92.21%, 82.82% and 88.51%, respectively, was observed under hypoxic conditions. Indicating the structural breakdown of azobenzene linkers in response to low levels of oxygen promoting the release of encapsulated drugs from HACS NPs, along with a decrease in average size from 108.08 ± 6.67 to 10.56 ± 4.98 nm, was noticed after 24 h of incubation in a low oxygen environment supporting the systems capability of controlled drug release and stability.

**FIGURE 8:**
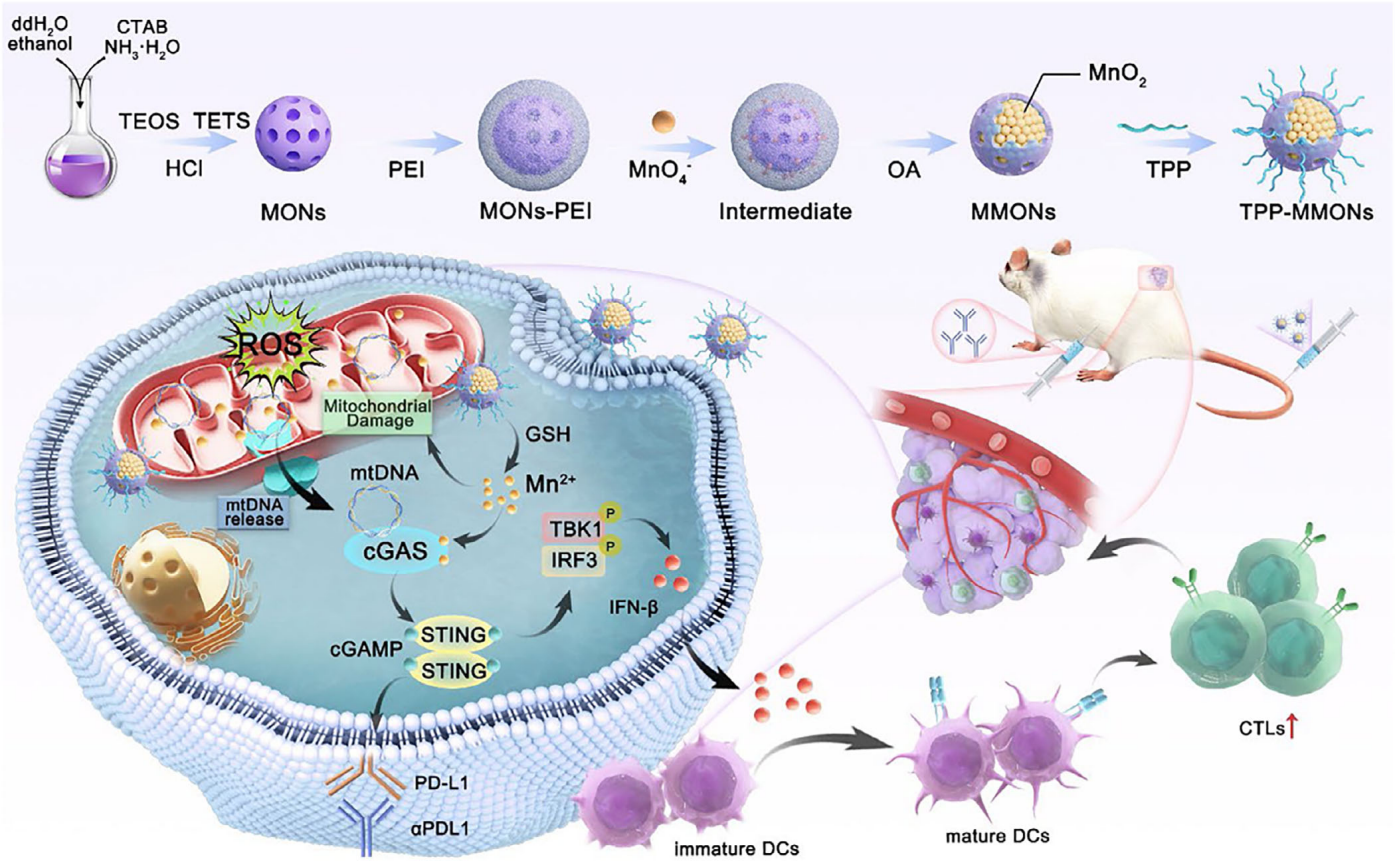
Schematic illustration of triphenyl‐phosphine (TPP)‐mesoporous organosilica nanoparticles (MMONs) nanoplatform‐induced activation of stimulator of interferon genes (STING) pathway based on the release of mitochondrial DNA (mtDNA) to enhance tumour immunotherapy.^61^

HA‐SS‐CPT@Ce6 NPs boost the maturation of bone marrow‐derived dendritic cells and initiate the STING cascade by activation of antigen‐presenting cells and T cells. Pharmacokinetic studies visualized an effective tumour accumulation of HACS NPs in 4T1 tumour bearing mouse model. Upon laser irradiation on nanoparticles, suppression in tumour growth was observed. The results open array for enhancing the therapeutic outcomes of surgical modalities such as HIFU (Figure 8).^63^

In a parallel strategy in the pursuit of targeted chemo‐immunotherapeutic strategy for breast cancer, Song et al. reported a cobalt(III) cyclam‐based prodrug (Co_2_) utilizing a dual modality approach jamming up immune activation by cCAS‐STING pathway with chemotherapy. The prodrug remains inert in normal physiological conditions and gets activated in response to altered acidic pH of TME, releasing both cytotoxic Co(II) ions and the STING agonist MSA‐2. The dual release causes DNA damage and activation of STING pathway leading to infiltration of IFN‐β, IL‐6 and IL‐12 in response to phosphorylation of TBK1 and IRF3. Co_2_ formulated into disulphide‐linked polymeric NP enhanced TNBC tumour targetability and pharmacokinetics enabling pronounced tumour suppression compared to cisplatin in 4T1 murine mouse model. An increase in M1/M2 macrophage ratio and reduction of myeloid derived suppressor cells were observed. In vitro assay revealed potent cytotoxicity of cobalt(III) cyclam‐based prodrug with low micromolecular IC_50_ values across MCF‐7, MDA‐MB‐231 and 4T1 breast cancer cell lines surpassing both monotherapy with MSA‐2 and cisplatin. Upon encapsulation within the disulphide‐linked polymeric NP, 57% of tumour inhibition was observed in 4T1 murine mice model surpassing cisplatin efficacy (36%), along with modulation of TME. PD‐1 upregulation on T cells enabled tumour eradication in synergy with anti‐PD‐1 checkpoint blockades, prolonging survival.^64^

Activation of STING pathway demonstrates a significant potential for amplifying antitumour immune response; however, systemic delivery of STING agonist may provoke off‐target proinflammatory response and diminished immune responsiveness. In order to mitigate these constrains, Ning et al. introduced an innovative phototheranostic strategy to activate NIR‐II‐regulated release of non‐nucleotide STING agonist MSA‐2. A pyridinium rotor‐based design was employed to develop a positively charged TBTP‐Bz co‐loaded with MSA‐2 into a thermally responsive exosome–liposome hybrid nanoparticle for tumour targeting. Upon laser irradiation 808 nm, TBTP‐Bz induces immunogenic cell death, and HMGB1 translocation is marked by ATP release and activation of STING pathway. This promotes infiltration of TNF‐α, IFN‐β and IL‐6 promoting maturation of dendritic cells. Among various intermolecular forces, TBTP‐Bz shows minimal electrostatic interaction (−534.79 kJ/mol) due to electronic repulsion of the positively charged pyridinium moiety, along with a high zeta potential of 62.0 mV ensuring its colloidal stability and packing. This streamlined image directed photoimmunotherapy enabling activation of STING pathway in poorly immunogenic TNBC. Justifying tumour suppression and sustained immunological cellular response.^65^

Cryoablation has emerged as a minimal invasive, cost‐effective technique for treatment of solid tumour. It involves insertion of a cryoprobe into solid tumour under image guidance followed by rapid freeze–thaw cycles exposing cells to temperature below −40°C. This rapid decrease in temperature causes formation of ice crystals leading to physical distortion of tumour cells. Beyond this, its magic doesn't pause here; it triggers a systemic immunogenic response termed abscopal effect targeting distant metastatic lesions. But the clinical translation of abscopal effect for treatment is limited due to the immunosuppressive tumour microenvironment. In order to address this challenge, Wang et al. developed novel nanoconstruct Ti3C2@Au‐PEG‐DMXAA featuring gold nanoparticles functionalized surface‐modified MXene (Ti3C2) nanosheets. These nanosheets loaded with classic non‐nucleotide STING agonist DMXAA were pegylated to enhance bioavailability. The attached gold nanoparticles enabled CT‐guided precise delivery of photothermal and immunomodulatory agents in solid immunosuppressive tumours. The combined effect of mild photothermal therapy and activated STING pathway generates strong immunogenic response repolarizing tumour‐associated macrophages, increasing cytotoxicity and stimulating dendritic cells transforming TME. This ignites the cryoablation effect eradicating local and distant metastatic tumour by releasing tumour‐associated antigens in situ. It induces long‐term immune memory, mitigating the risk of tumour relapse and metastatic progression.^62^

In a significant stride towards multimodal cancer therapy, integrating chemotherapy, sonodynamics and induction of immune response by activation of cGAS‐STING pathway represents a promising clinical and therapeutic approach. This combinatorial approach helps to overcome the key limitations of conventional therapy, including limited therapeutic efficiency, drug resistance and other physicochemical constraints in drug delivery. Adding more this, Pu et al. developed an ultrasound responsive cRGD‐modified nanobubble co‐loaded with paclitaxel and sonosensitizer [chlorine e6 (Ce6)] modified lipid. This helped to achieve targeted imaging, cRGD‐targeted tumour delivery, ultrasound‐triggered ROS generation and payload release while simultaneously overcoming the limited drug loading capacity inherent to monolayer lipid shells in conventional ultrasound contrast agents. Upon activation, the 500 nm‐sized nanobubble along with submicron nanobubble (100–1000 nm) accumulated by EPR effect release their payload in a controlled release fashion promoting immunogenic cell death by cGAS‐STING activation and mitotic disruption by PTX. The modified DSPE‐PEG‐Ce6 lipid facilitates real‐time imaging on internalization.

**FIGURE 9:**
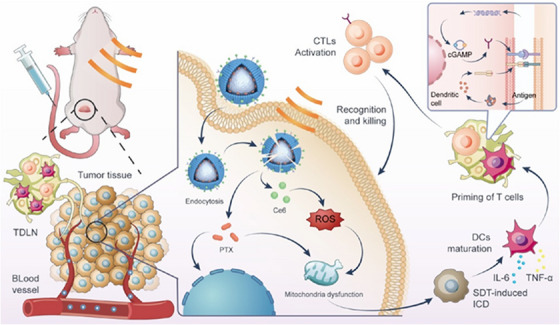
Schematic illustration of Ce6/PTX Nb‐mediated SDT‐responsive sonoimmunotherapy.^66^

Expanding upon these, the findings of dual‐mode imaging indicate that Ce6/PTX nanobubbles confer potent tumour targetability and enhanced bioavailability validating the therapeutic relevance of sonodynamics. In vitro findings suggest a significant reduction in cell viability <50% upon optimization of therapeutic ratio of 1:3 of PTX/Ce6 in Ce6/PTX Nbs. Mechanistically, this therapeutic configuration also marked an upregulation of p‐STING and p‐IRF3 expression in dendritic cells as conferred by western blotting. A significant tumour suppression marked by reduction in weight of Ce6/PTX Nbs administered to 4 T1 mouse model with metastatic inhibition up to 93.9%, surpassing the outcomes achieved by monotherapy. Ki‐67 staining indicates reduction in proliferation and cell necrosis. Overall, the study exemplifies a next‐generation theranostic offering a clinical translation for breast cancer and other immunosuppressive malignancies (Figure 9).^66^

### 1.8 Challenges and future perspectives

STING‐cascade‐mediated NPs offer a promising approach to reprogramming the immunosuppressive TME by leveraging the heterogenous tumour biology of TNBC. Despite the promising antitumour efficacy demonstrated in preclinical models, STING agonists and natural cyclic nucleotides like cGAMP are prone to rapid degradation by ectonucleotide pyrophosphate/phosphodiesterase 1. This phosphodiesterase promptly diminishes their biological half‐life in systemic circulation, thereby challenging their pharmacokinetic stability, penetrability and off‐targeting. However, multimodal NPs help to overcome these limitations by shielding it from direct physiological interaction during circulation while simultaneously enhancing the drug loading and encapsulation efficiency of hydrophilic STING agonists.^67^, ^68^ Nanotherapeutics that rely on the enhanced permeability and retention effect for delivery face limitations in delivery due to heterogenicity of TNBC TME, elevated levels of interstitial fluid pressure and increased stromal density.^41^, ^69^, ^70^

Furthermore, the reduction of oxygen level in TME due to rapid angiogenesis initiates hypoxia‐induced metabolic reprogramming. This competitively inhibits the STING cascade by accumulation of ADSL‐mediated fumarate affecting the therapeutic efficacy.^71^, ^72^ To overcome all these challenges, next‐generation nanocarriers are being developed by integrating stimuli‐responsive, logic gate systems, biomimetics, cell membrane coated and manganese‐based NPs.^1^ They activate the STING cascade through multiple cell death mechanisms like ferroptosis, apoptosis and cuproptosis.^73^, ^74^

## 2 CONCLUSION

TNBC remains one of the most aggressive subtypes of breast cancer due to its immunological complexity, the absence of hormone receptors and high rate of metastasis. The STING pathway is emerging as critical pathway translating the immunogenic ‘cold’ TNBC tumour into ‘hot’ one, thereby improving the responsiveness to immune checkpoint blokade.

Even though the significant barriers still exist, including limited tumour penetrability, rapid degradation of natural STING agonists and TME‐induced metabolism. Recent advances in the multimodal therapy circumvent these by multiple cell apoptotic pathways (ferroptosis/cuproptosis/mitochondrial DNA disruption) initiating the infiltration of type 1 interferons activating the STING cascade.

The advancements of STING‐cascade activating NPs mark a transformatory step in treatment of TNBC. They not only address the complex challenges associated with the most aggressive subtype of BC but also enhance the immunoresponsive. The review visualizes the clinical translation of mind maps into clinical reality.^75^, ^76^

To accelerate, the upcoming research clinicals should priorities long‐term adaptive immunity, optimizing bioavailability and enhancement of tumour targetability. Ultimately, the integration of immunology, nanotechnology and targeted therapy activating STING cascade transforms TNBC therapy.

## AUTHOR CONTRIBUTIONS

Harshita Singhai, Taha Alqahtani, Humood Al Shmrany and Garima Gupta wrote the manuscript. Umesh Kumar Patil, Amirhossein Sahebkar and Prashant Kesharwani conceptualized, proofread and supervised during writing of the original manuscript. All authors reviewed the manuscript.

## CONFLICT OF INTEREST STATEMENT

The authors declare no conflicts of interest.

## ETHICS STATEMENT

Not applicable.

## Data Availability

Not applicable.
